# Supplementary material for: Differential V2-directed antibody responses in non-human primates infected with SHIVs or immunized with diverse HIV vaccines
Source: Nat Commun. 2022 Feb 16;13:903. doi: 10.1038/s41467-022-28450-1 (PMC8850611; doi:10.1038/s41467-022-28450-1)
Supplement: Supplementary file 1 — Supplementary Information [file 41467_2022_28450_MOESM1_ESM.pdf]

Supplementary Figure S1a

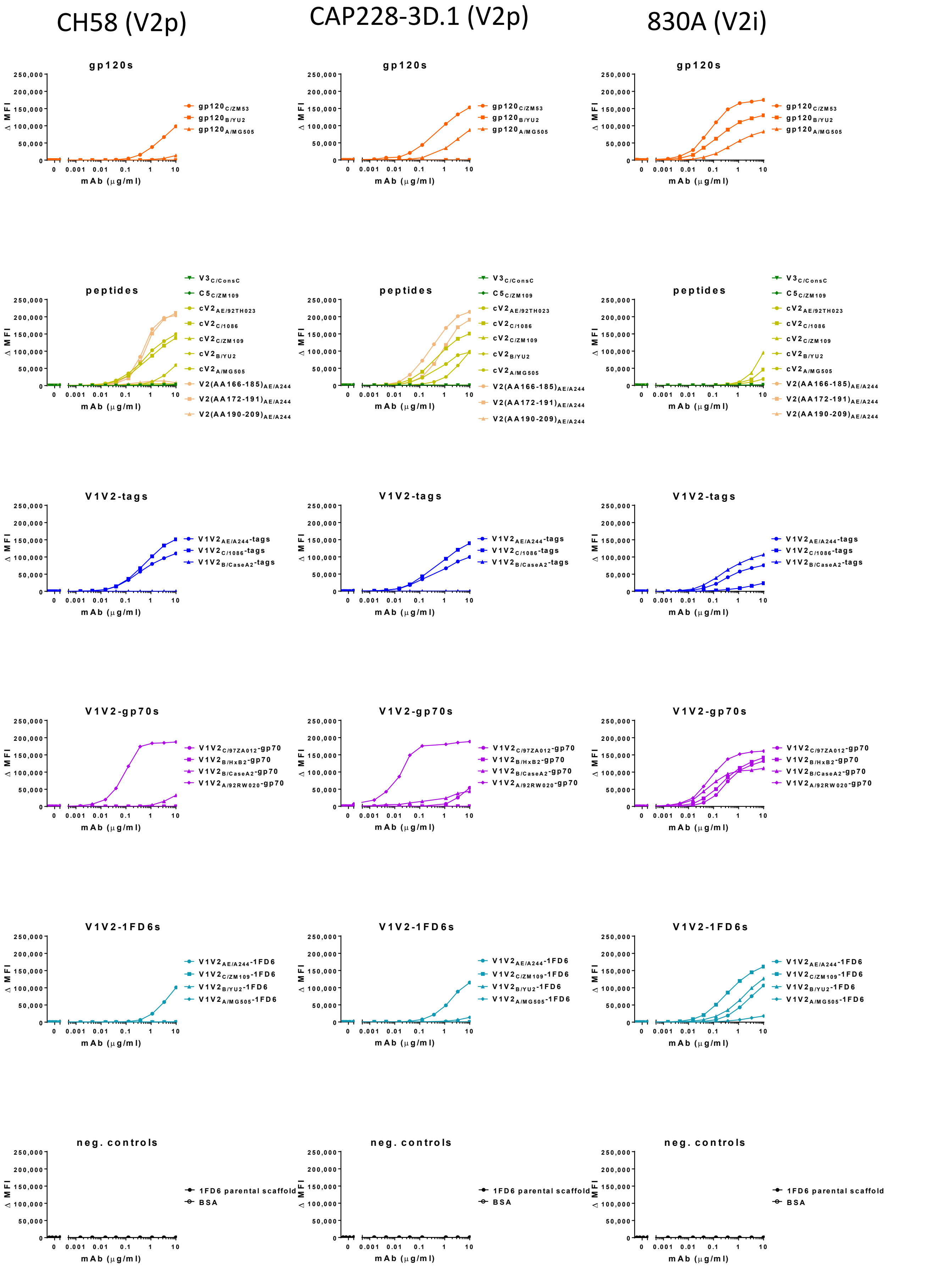

Supplementary Figure 1b

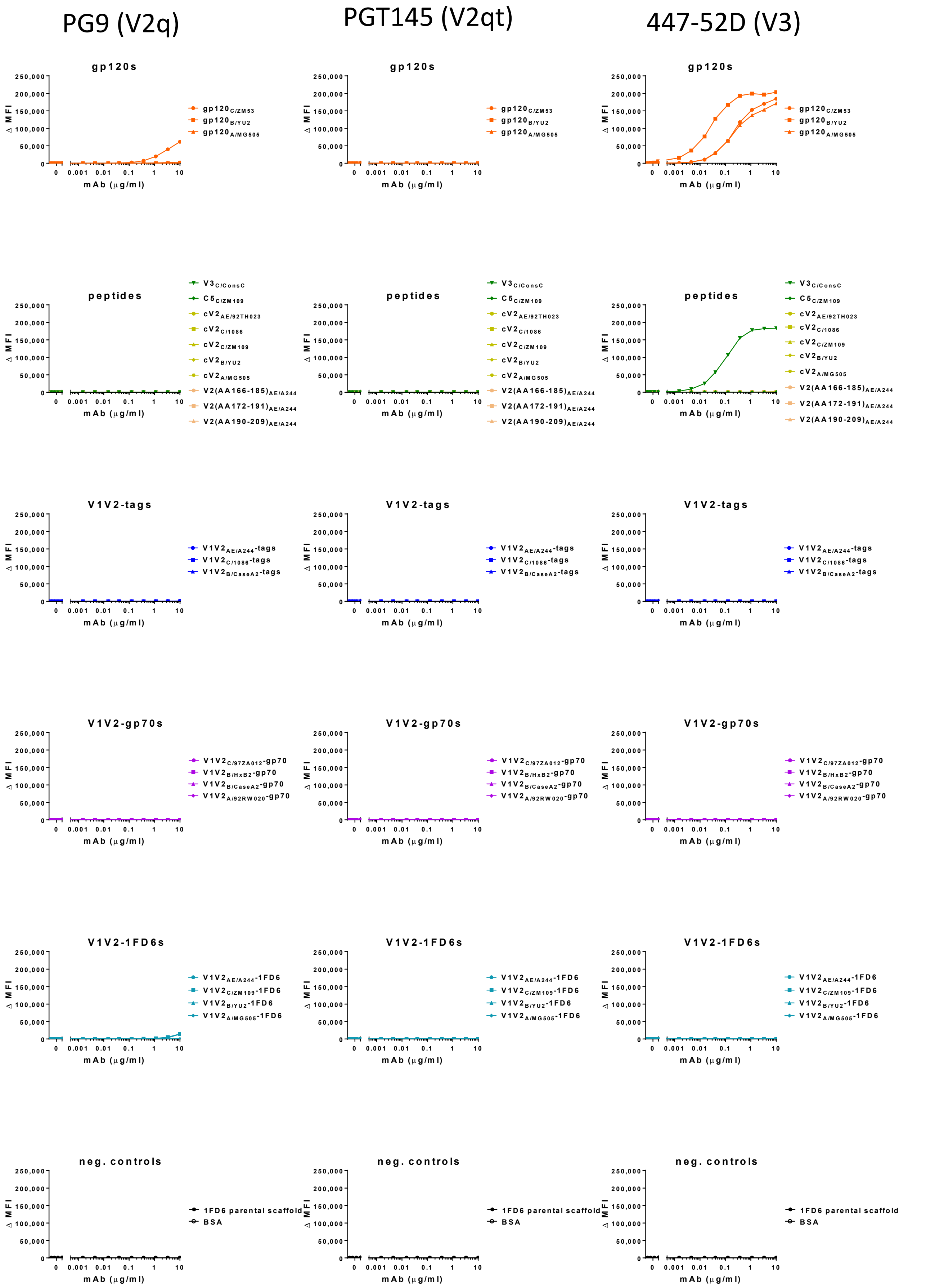

Supplementary Figure S1. Titration of V2 mAbs with HIV-1 Env antigens. Using the multiplex bead Ab binding assay, the following mAbs were titrated for reactivity with antigens used in Figure 1a. Panel a: CH58 and CAP228-3D.1 (specific for V2p [column 1 and 2]), 830A (specific for V2i [column 3]), Panel b: PG9 (specific for V2q [column 1]), PGT145 (specific for V2qt [column 2]) and 447-52D (specific for V3 [column 3]). Antigens include gp120s (orange), peptides (green and cream), V1V2-tags (blue), V1V2-gp70s (purple), V1V2-1FD6s (cyan). Parental 1FD6 scaffold and BSA (black) were used as negative controls. Data are shown as ΔMFI calculated from experiments normalized on the basis of the reactivity of a mAb pool from which background (PBS-TB) was subtracted. Source data are provided as a Source Data file.

Clade	Antigen	mAb	CH58	CH59	CAP228 19F	CAP228 3D.1	CAP228 16H	830A	697-30D	2158	1361	PG9	PGT145	447-52D
		Specificity	V2p					V2i				V2q	V2qt	V3
AE	A244/92TH023	cV2	5.70	6.51	6.20	4.63	6.68	0.37	0.04	0.01	0.01	0.00	0.00	0.01
		V2(AA166-185)	9.21	9.73	10.26	9.64	10.73	0.01	0.10	0.00	0.00	0.00	0.00	0.04
		V2(AA172-191)	9.11	9.29	10.31	8.00	11.23	0.01	0.04	0.00	0.00	0.00	0.00	0.03
		V2(AA190-209)	0.57	0.90	0.19	0.05	0.22	0.01	0.05	0.00	0.00	0.00	0.00	0.03
		V1V2-tags	4.13	5.45	3.97	4.21	4.00	3.32	0.40	2.32	1.72	0.03	0.00	0.00
		V1V2-1FD6	3.18	3.35	5.20	4.30	6.76	3.82	2.64	2.82	2.63	0.32	0.00	0.01
C	ZM109	C5	0.13	0.04	0.07	0.04	0.15	0.03	0.02	0.02	0.03	0.00	0.00	0.01
		cV2	0.09	8.32	0.03	0.01	0.06	2.48	0.06	0.00	2.79	0.00	0.00	0.03
		V1V2-1FD6	0.01	0.01	0.00	0.01	0.01	7.06	0.03	6.40	7.60	0.37	0.00	0.00
	1086	cV2	6.69	7.79	7.89	6.50	7.66	1.23	0.63	0.10	0.03	0.00	0.00	0.03
		V1V2-tags	6.05	7.29	7.18	5.88	7.02	0.83	0.01	0.06	0.79	0.00	0.00	0.00
	ZM53	gp120	3.18	0.00	7.22	6.46	6.94	6.66	5.06	5.86	6.19	2.08	0.00	6.67
	consC	V3	0.12	0.03	0.02	0.02	0.07	0.07	0.15	0.04	0.04	0.00	0.00	8.90
	97ZA012	V1V2-gp70	0.01	0.69	7.63	1.52	7.07	5.87	5.82	6.31	6.57	0.00	0.00	0.00
B	YU2	gp120	0.01	0.01	0.02	0.00	0.02	5.49	3.55	4.06	3.93	0.04	0.00	9.82
		cV2	0.26	0.35	0.14	0.05	0.18	0.01	0.07	0.00	0.00	0.00	0.00	0.03
		V1V2-1FD6	0.04	0.04	0.03	0.03	0.03	4.90	3.70	4.45	4.69	0.00	0.03	0.03
	CaseA2	V1V2-tags	0.01	0.00	0.23	0.01	0.21	4.72	3.92	4.37	4.59	0.00	0.00	0.00
		V1V2-gp70	1.04	0.00	3.51	1.80	3.74	5.27	4.35	5.43	5.58	0.00	0.00	0.00
	HxB2	V1V2-gp70	0.01	0.00	0.78	0.01	0.57	6.34	5.61	6.08	7.45	0.00	0.00	0.00
A	MG505	gp120	0.45	0.01	3.95	3.11	3.64	3.52	0.67	1.47	1.58	0.11	0.00	7.62
		cV2	1.73	0.24	4.81	3.14	6.99	0.00	0.13	0.00	0.00	0.00	0.00	0.02
		V1V2-1FD6	0.03	0.00	0.18	0.38	0.19	0.63	0.00	0.00	0.01	0.00	0.00	0.00
	92RW020	V1V2-gp70	8.87	3.30	9.01	9.23	9.23	7.78	0.05	4.68	6.53	0.00	0.00	0.00

Clade:
A
B
C
E

Binding in relative AUC

15-10	very strong
10-5	strong
5-1	moderate
1-0.5	weak
0.5-0	no

Supplementary Figure S2. Reactivity of HIV-1 Env antigens with V2 monoclonal antibodies, sorted by clade and strain. These data are resorted from those shown in Figure 1 where a multiplex bead binding assay was used to determine the levels of reactivity of mAbs specific for different V2 epitopes with various HIV-1 antigens. The clade from which each antigen was derived is indicated by the subscripted letter and by color coding. As a control, V3 mAb 447-52D was used. Irrelevant mAbs and PBS were used as negative controls in each experiment (not shown). Data are shown as AUC values generated from titration curves for each mAb, provided in Supplementary Figure S1. Strength of binding is color-coded as per the spectrum shown in the figure. Experiments were performed at least twice.

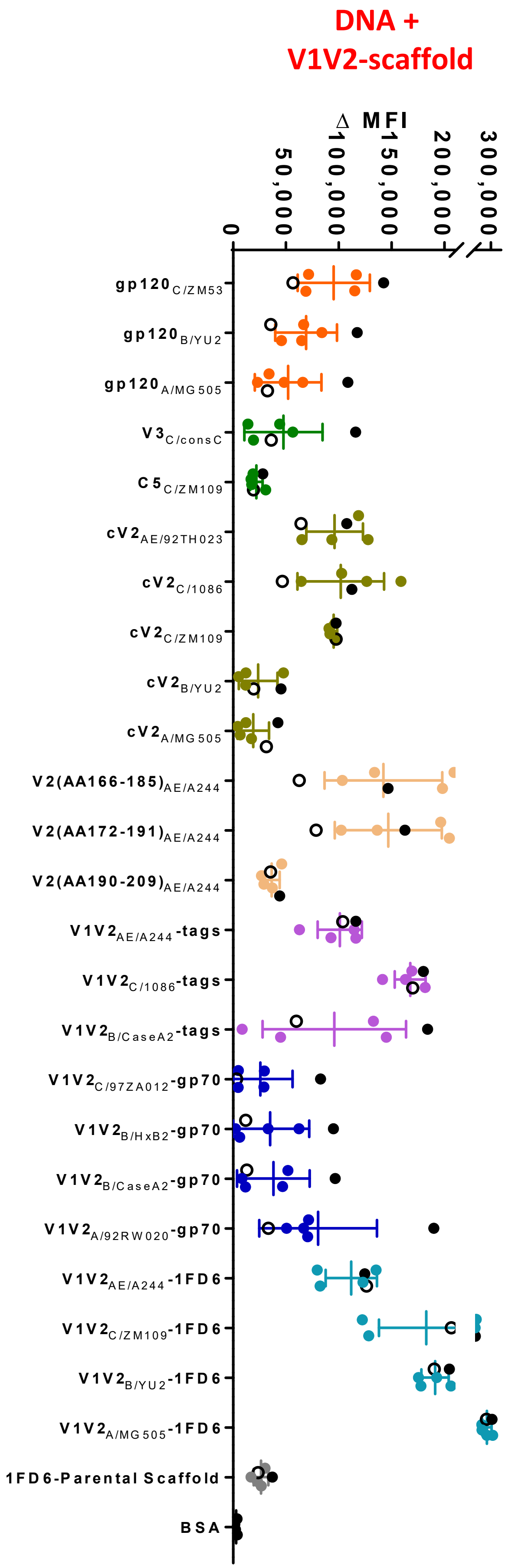

Supplementary Figure S3. Dot plot of data from the NHPs immunized with the "DNA + V1V2-scaffold" regimen. Data derived from Figure 3 and Supplementary Figure S4 are displayed here to show the relative activities of the animal responding most strongly (NHP 31444, ●) and the animal responding most weakly (NHP 29251, ○) to gp120s. Responses of these animals to all antigens are consistently strong and weak, respectively, relative to other animals receiving the same regimen. Mean and the standard deviation (SD) are depicted.

Supplementary Figure S4a

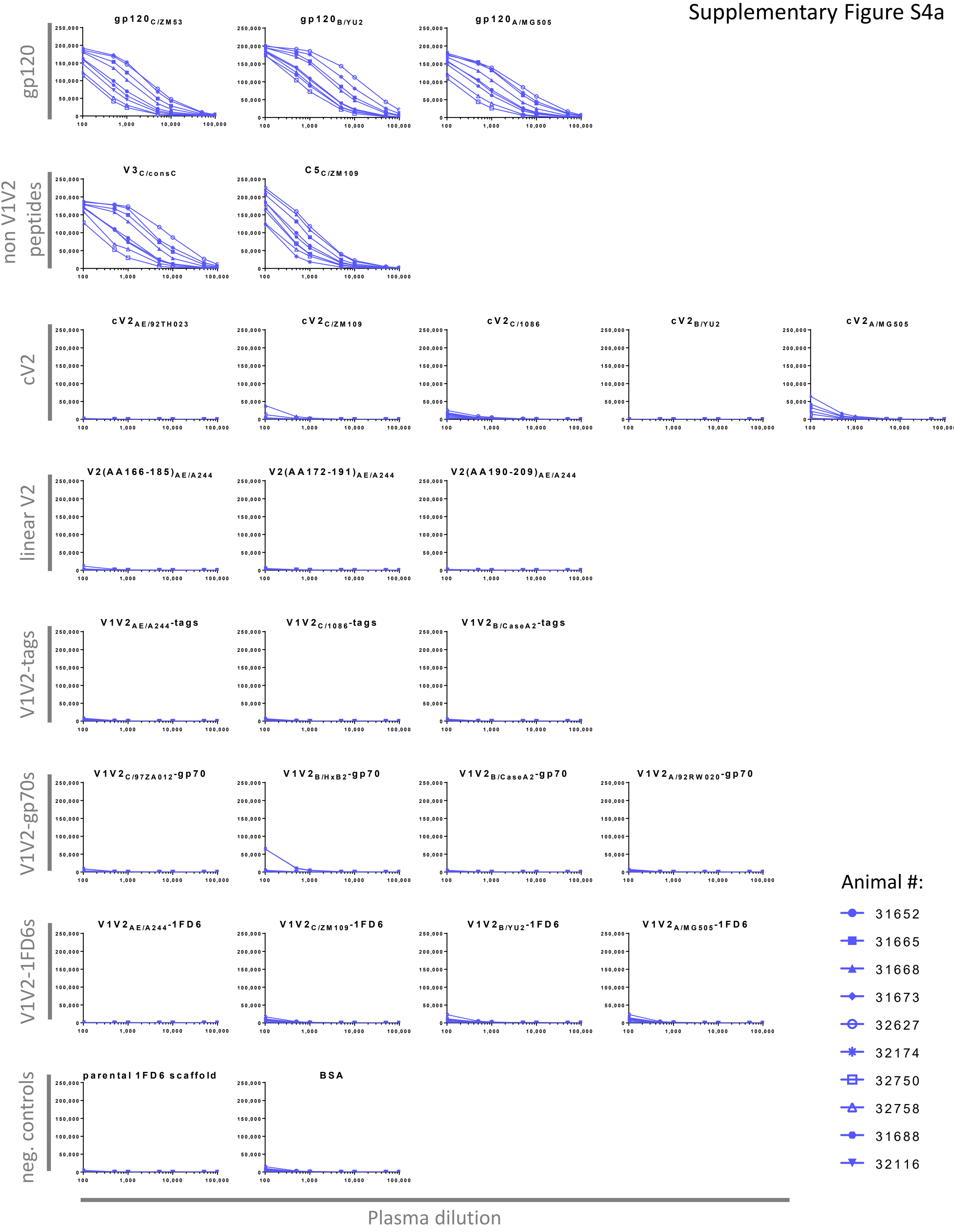

Supplementary Figure S4b

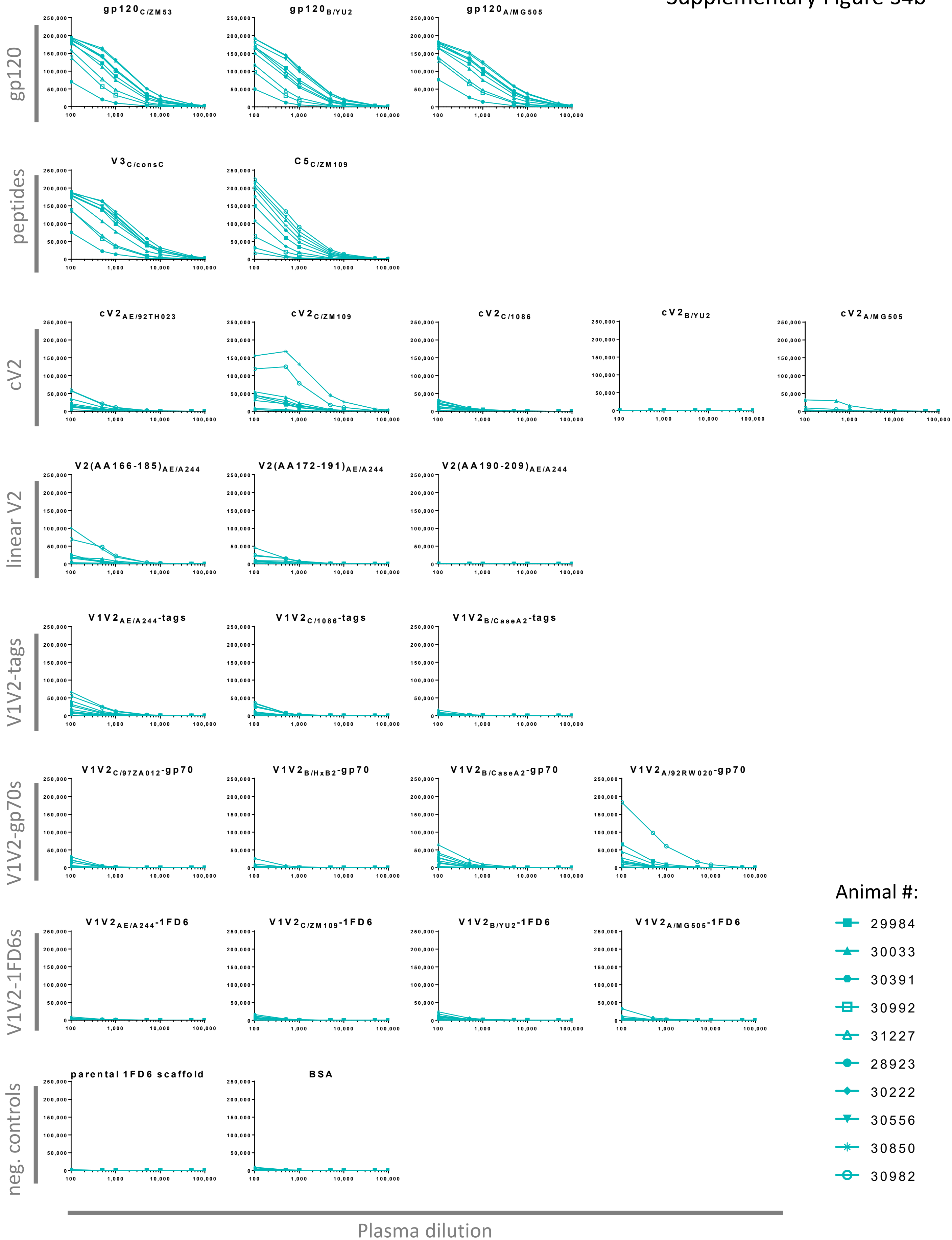

Supplementary Figure S4c

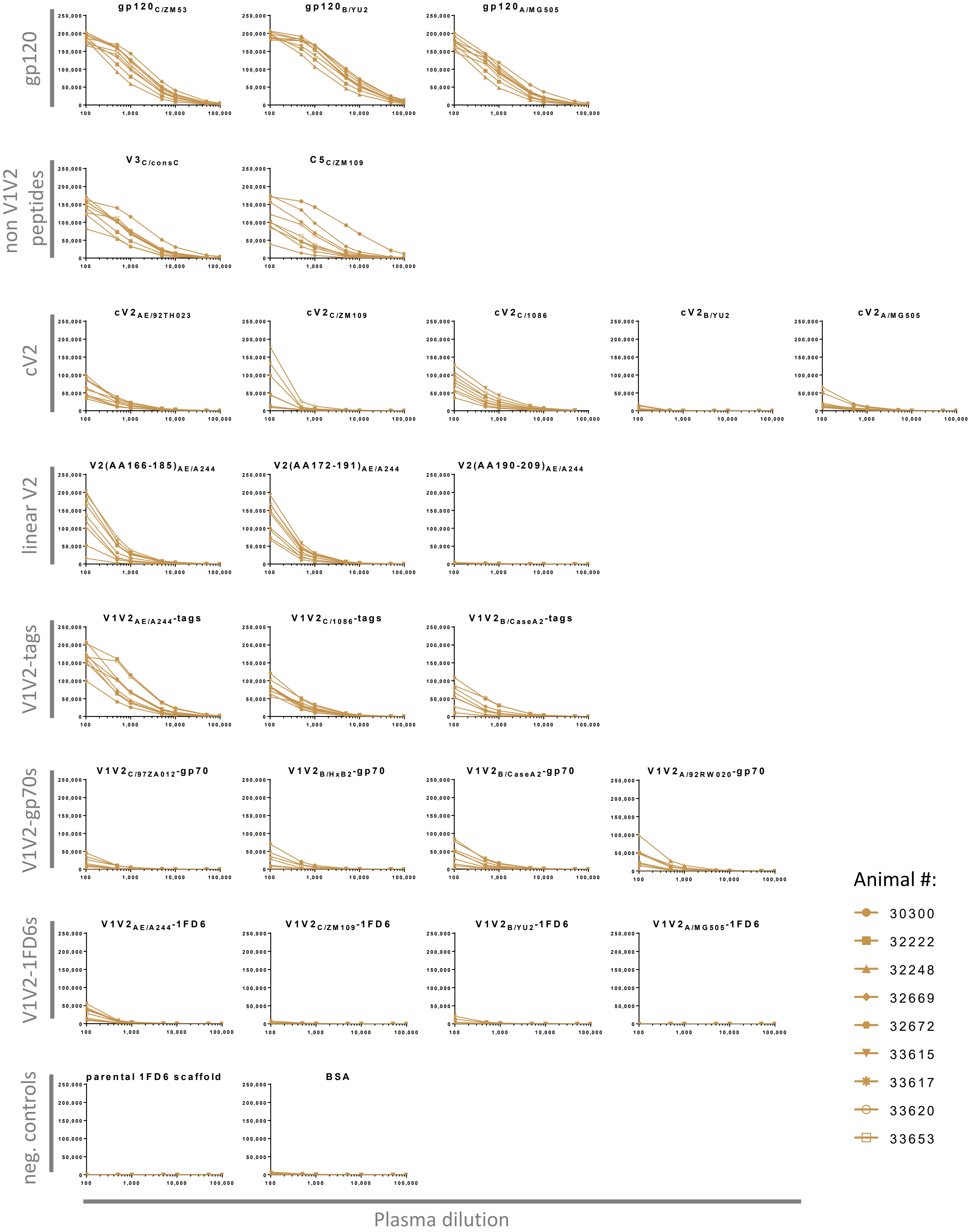

Supplementary Figure S4. Plasma from infected or immunized NHPs titrated for binding to Env Ags. Plasma were titrated from 1:100 – 1:150,000. a Plasma samples drawn at 11 weeks post last challenge from NHPs infected with clade C Tier 2 $SHIV_{1157\text{-}ipd3N4}$ (n=10). b Plasma samples drawn at 18 weeks post last challenge from NHPs infected with clade C Tier 1 $SHIV_{1157ipEL\text{-}p}$ (n=10), and c plasma from NHPs immunized with "DNA + gp120" (n=9) drawn two weeks after the last immunization. Identical antigens were used in all panels and are designated by category on the left and delineated on each graph. Data are shown as ΔMFI calculated from experiments normalized on the basis of the reactivity of the mAb pool from which background (PBS-TB) was subtracted. Two to four independent experiments were performed, of which one representative is shown. Source data are provided as a Source Data file.

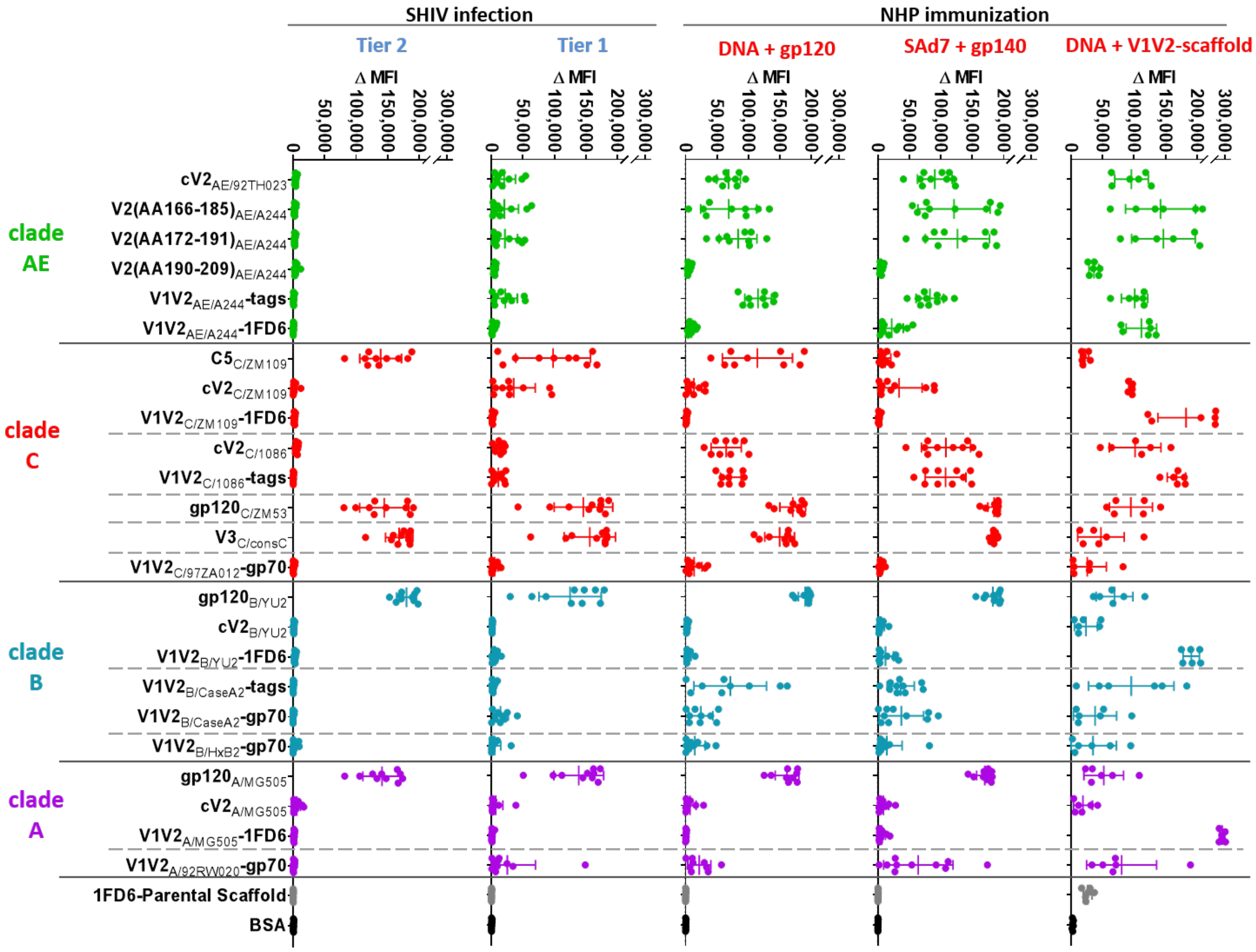

Supplementary Figure S5. Dot plot of Ab binding activities in plasma from infected and immunized NHPs assessed in the multiplex bead Ab binding assay. These data are resorted from those shown in Figure 3 with the vertical groupings shown by clade and strain rather than by antigen type (peptide, gp120, etc.). Data columns from left to right: Tier 2 $SHIV_{C/1157-ipd3N4}$-infected NHPs (n=10); Tier 1 $SHIV_{C/1157ipEL-p}$-infected NHPs (n=10); NHPs immunized with "DNA + gp120" (n=9); NHPs immunized with "SAd7 + gp140" (n=10); NHPs immunized with "DNA + V1V2-scaffolds" (n=6). Each data point is derived from a biologically independent plasma specimen. Binding activity of plasma was measured against the same set of 24 Env antigens used in Figure 1a. Beads identified as "1FD6 parental scaffold" (devoid of the V1V2 insert) and BSA (bovine serum albumin) were used as (ΔMFI) generated with negative controls. Each dot represents the normalized MFI of a plasma specimen diluted 1:200 from a single animal. Bars indicate mean and standard deviation (SD). Plasma samples from infected NHPs were drawn 18 and 11 weeks post last challenge for Tier 1 SHIV- and Tier 2 SHIV-infected NHPs, respectively. Specimens from immunized NHPs were drawn two weeks after the last immunization. A mAb pool was used as a positive Ab control (not shown). Intensity of the reactivity is shown as the mean MFI calculated from experiments normalized on the basis of the reactivity of the mAb pool in each experiment from which background (PBS-TB) was subtracted. Experiments were performed at least twice and in each experiment, samples were tested in duplicate.

Supplementary Figure S6a

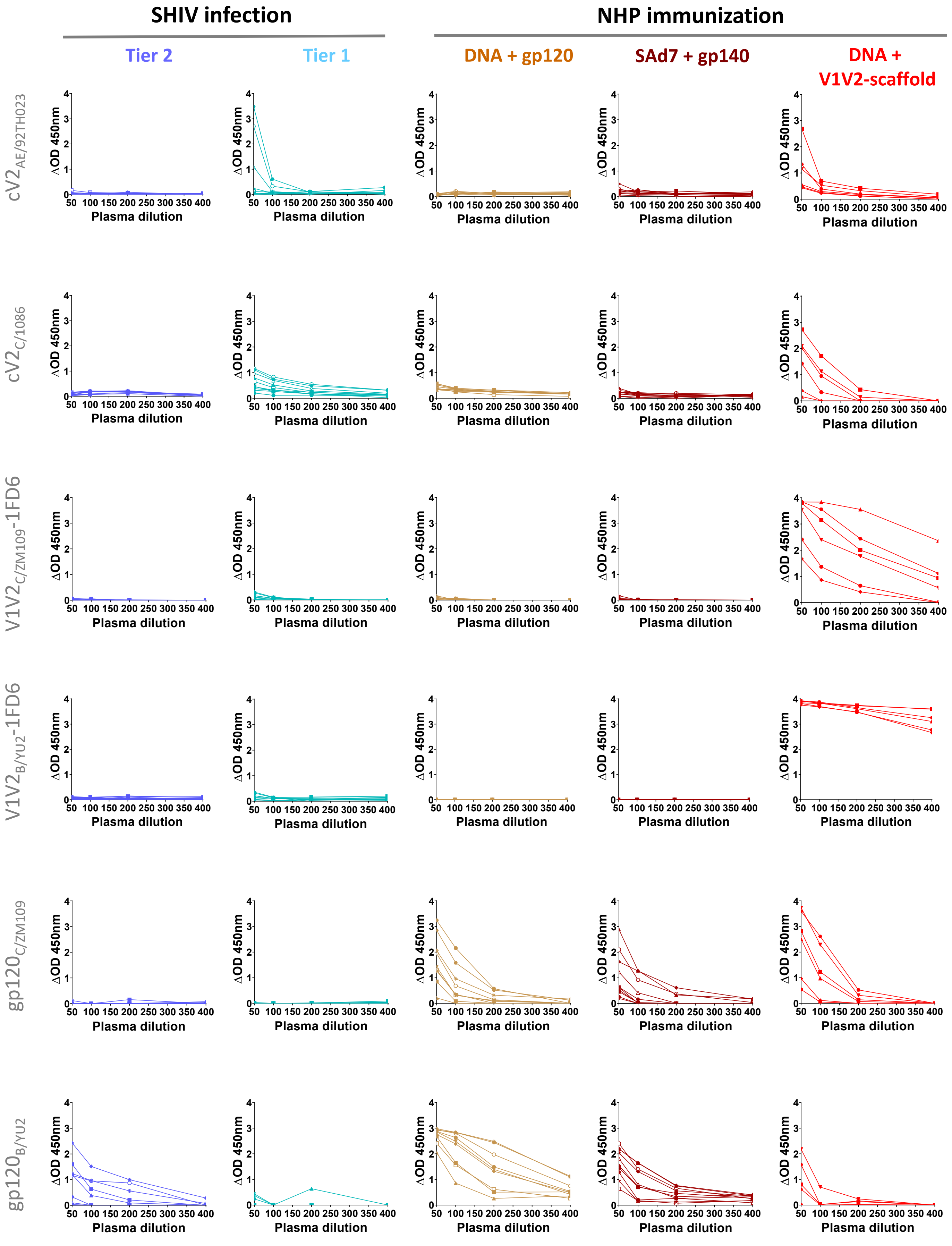

Supplementary Figure 6b

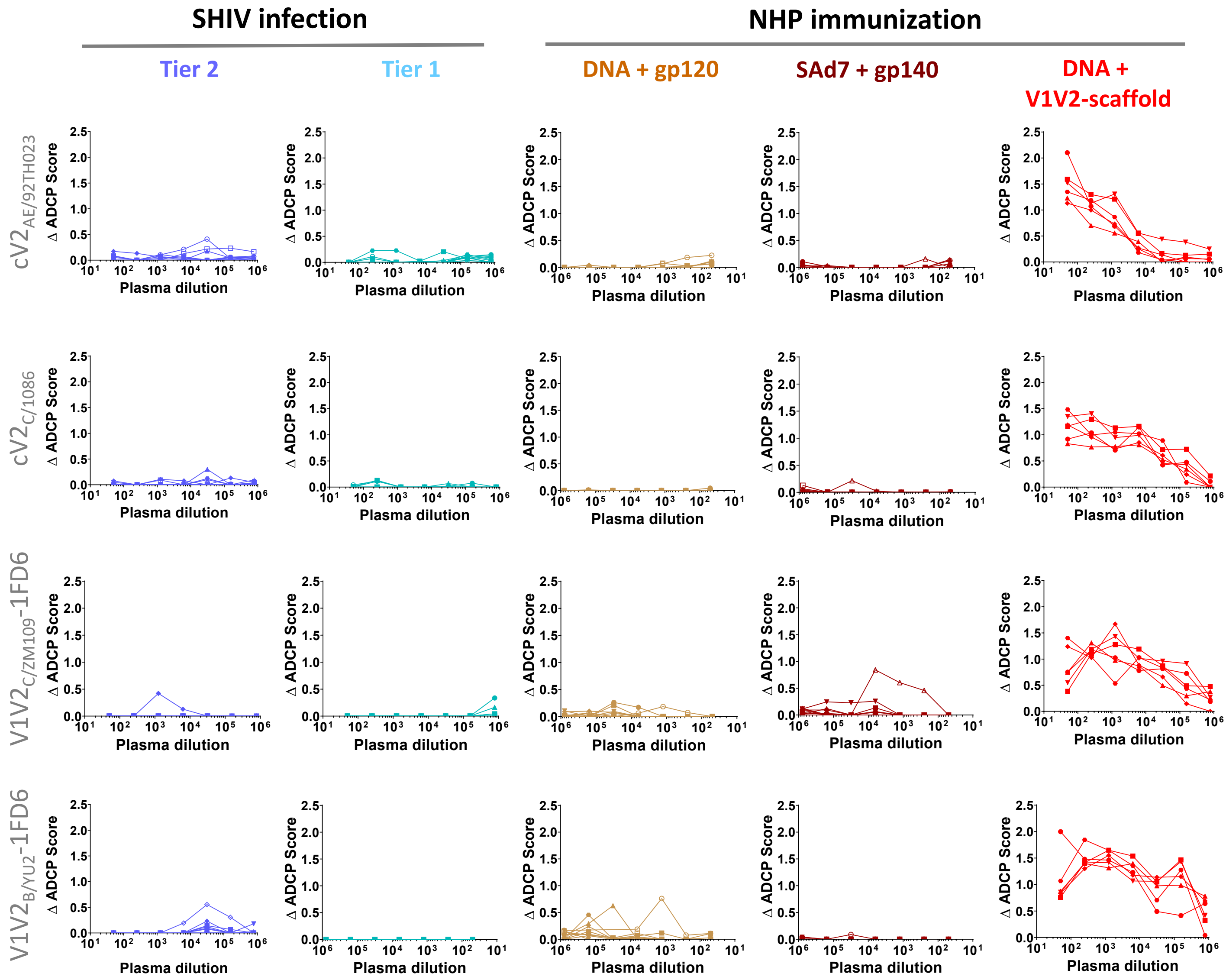

Supplementary Figure S6. Titration curves showing C1q binding and ADCP responses of SHIV-infected NHPs compared to immunized NHPs. a C1q binding to V2p Abs was measured using cV2 peptides as antigens, and C1q binding to V2i-Abs was measured using V1V2-1FD6 antigens. C1q binding to Abs against all Env epitopes was measured using gp120 from two HIV-1 strains. Plasma samples were titrated two-fold from 1:50 - 400. C1q data represent a single experiment. b ADCP mediated by V2p Abs was measured using cV2 peptides as antigens, and ADCP mediated by V2i Abs was measured using V1V2-1FD6 antigens. NHP plasma were titrated as five-fold dilutions ranging from 1:50 - 781,250. ΔADCP scores were calculated by multiplying the percentage of bead-positive cells by mean fluorescent intensity (MFI) and subtracting the same product obtained with pre-bleeds followed by division with a denominator giving values between 0 and 10. Plasma used for each NHP group was 11 weeks and 18 weeks after the last challenge with SHIV Tier 2 or SHIV Tier 1, respectively, and two weeks after immunization. ADCP experiments were performed at least twice. Source data are provided as a Source Data file.

a

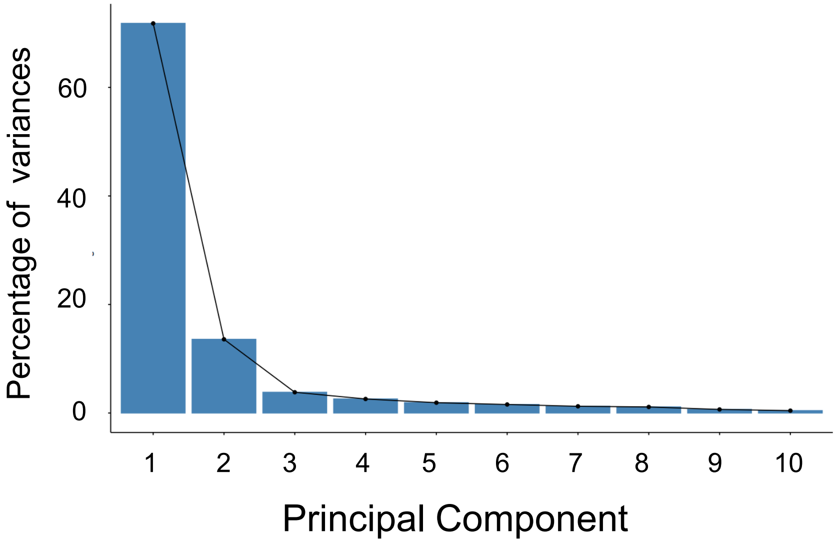
Percentage of variances
60
40
20
0
1
2
3
4
5
6
7
8
9
10
Principal Component

b

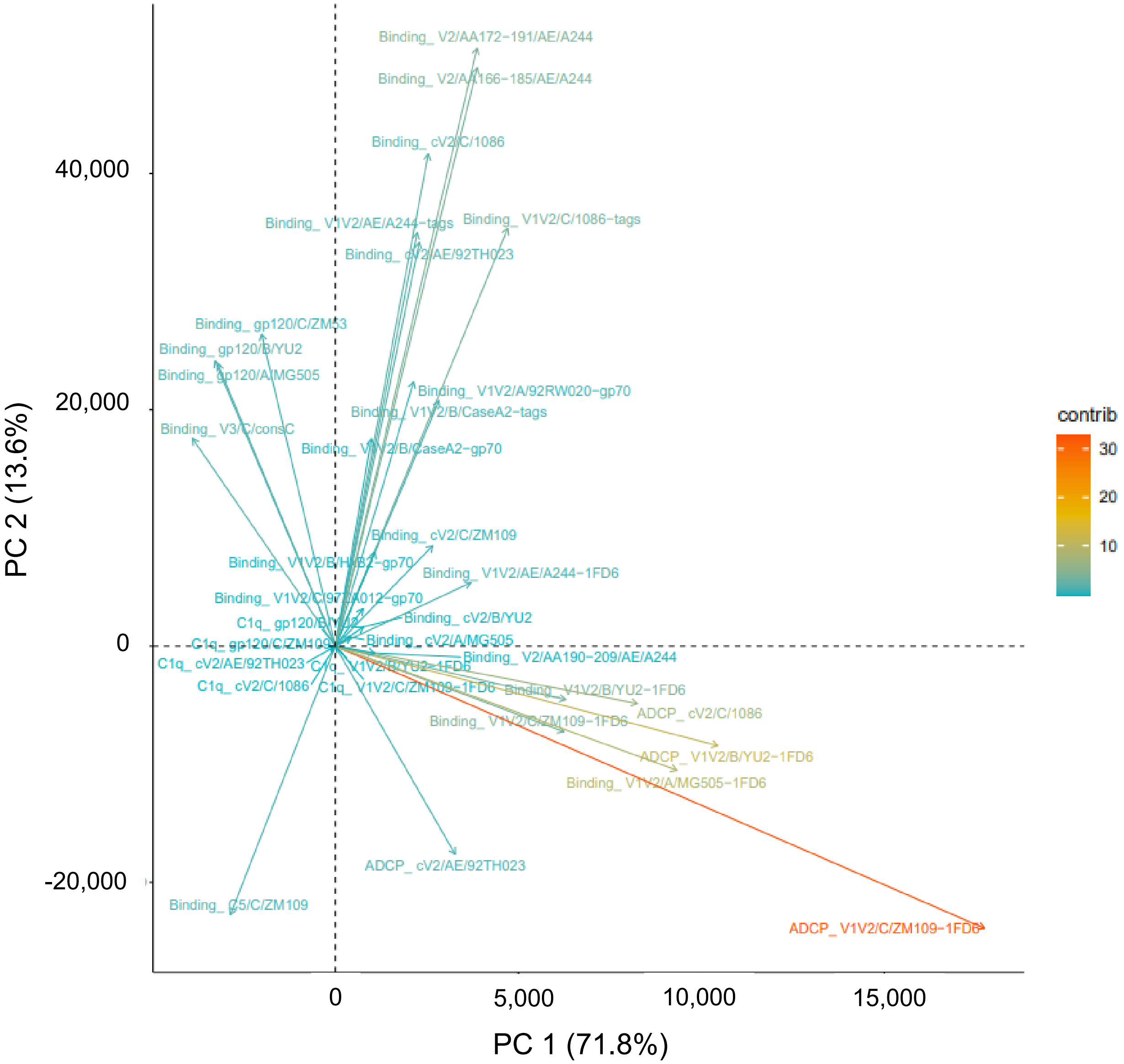
Binding_ V2/AA172-191/AE/A244
Binding_ V2/AA166-185/AE/A244
Binding_ cV2/C/1086
Binding_ V1V2/AE/A244-tags
Binding_ V1V2/C/1086-tags
Binding_ cV2/AE/92TH023
Binding_ gp120/C/ZM53
Binding_ gp120/B/YU2
Binding_ gp120/A/MG505
Binding_ V1V2/A/92RW020-gp70
Binding_ V1V2/B/CaseA2-tags
Binding_ V3/C/consC
Binding_ V1V2/B/CaseA2-gp70
Binding_ cV2/C/ZM109
Binding_ V1V2/B/HXB2-gp70
Binding_ V1V2/AE/A244-1FD6
Binding_ V1V2/C/97ZA012-gp70
Binding_ cV2/B/YU2
C1q_ gp120/B/YU2
Binding_ cV2/A/MG505
C1q_ gp120/C/ZM109
Binding_ V2/AA190-209/AE/A244
C1q_ cV2/AE/92TH023
C1q_ V1V2/B/YU2-1FD6
C1q_ cV2/C/1086
C1q_ V1V2/C/ZM109-1FD6
Binding_ V1V2/B/YU2-1FD6
ADCP_ cV2/C/1086
Binding_ V1V2/C/ZM109-1FD6
ADCP_ V1V2/B/YU2-1FD6
Binding_ V1V2/A/MG505-1FD6
ADCP_ cV2/AE/92TH023
Binding_ C5/C/ZM109
ADCP_ V1V2/C/ZM109-1FD6
contrib
30
20
10
40,000
20,000
0
-20,000
PC 2 (13.6%)
0
5,000
10,000
15,000
PC 1 (71.8%)

Supplementary Figure S7. Qualitative interpretation of principal component analysis. a Scree plot displaying the percentage of variances explained by the first ten principal components of the PC analysis shown in Figure 5b. b Loading plot showing the relationships between all variables in the space of the first two principal components PC 1 and PC 2. The color indicates total contribution of a given variable on explaining the variations retained by the first two principal components.

Supplementary Figure S8

			r	*p*	adjusted *p* Benjamini-Hochberg
**Cluster 1**	Binding_ gp120/B/YU2	Binding_ V3/C/consC	0.83	<0.0001	<0.0001
	Binding_ gp120/B/YU2	Binding_ gp120/C/ZM53	0.82	<0.0001	<0.0001
	Binding_ gp120/B/YU2	Binding_ gp120/A/MG505	0.89	<0.0001	<0.0001
	Binding_ V3/C/consC	Binding_ gp120/C/ZM53	0.83	<0.0001	<0.0001
	Binding_ V3/C/consC	Binding_ gp120/A/MG505	0.93	<0.0001	<0.0001
	Binding_ gp120/C/ZM53	Binding_ gp120/A/MG505	0.94	<0.0001	<0.0001
**Cluster 4**	Binding_ gp120/B/YU2	C1q_V1V2/C/ZM109/1FD6	-0.61	<0.0001	<0.0001
	Binding_ gp120/B/YU2	ADCP_V1V2/B/YU2/1FD6	-0.58	<0.0001	0.0001
	Binding_ gp120/B/YU2	ADCP_cV2/C/1086	-0.67	<0.0001	<0.0001
	Binding_ gp120/B/YU2	Binding_V1V2/AE/A244/1FD6	-0.56	0.0001	0.0002
	Binding_ gp120/B/YU2	Binding_V2/AA190/209/AE/A244	-0.60	<0.0001	<0.0001
	Binding_ gp120/B/YU2	Binding_V1V2/B/YU2/1FD6	-0.66	<0.0001	<0.0001
	Binding_ gp120/B/YU2	C1q_V1V2/B/YU2/1FD6	-0.69	<0.0001	<0.0001
	Binding_ gp120/B/YU2	Binding_V1V2/A/MG505/1FD6	-0.67	<0.0001	<0.0001
	Binding_ gp120/B/YU2	ADCP_V1V2/C/ZM109/1FD6	-0.69	<0.0001	<0.0001
	Binding_ gp120/B/YU2	Binding_V1V2/C/ZM109/1FD6	-0.65	<0.0001	<0.0001
	Binding_ gp120/B/YU2	ADCP_cV2/AE/92TH023	-0.62	<0.0001	<0.0001
	Binding_ gp120/B/YU2	Binding_cV2/C/ZM109	-0.45	0.0018	0.0034
	Binding_ V3/C/consC	C1q_V1V2/C/ZM109/1FD6	-0.66	<0.0001	<0.0001
	Binding_ V3/C/consC	ADCP_V1V2/B/YU2/1FD6	-0.76	<0.0001	<0.0001
	Binding_ V3/C/consC	ADCP_cV2/C/1086	-0.73	<0.0001	<0.0001
	Binding_ V3/C/consC	Binding_V1V2/AE/A244/1FD6	-0.68	<0.0001	<0.0001
	Binding_ V3/C/consC	Binding_V2/AA190/209/AE/A244	-0.72	<0.0001	<0.0001
	Binding_ V3/C/consC	Binding_V1V2/B/YU2/1FD6	-0.77	<0.0001	<0.0001
	Binding_ V3/C/consC	C1q_V1V2/B/YU2/1FD6	-0.80	<0.0001	<0.0001
	Binding_ V3/C/consC	Binding_V1V2/A/MG505/1FD6	-0.78	<0.0001	<0.0001
	Binding_ V3/C/consC	ADCP_V1V2/C/ZM109/1FD6	-0.78	<0.0001	<0.0001
	Binding_ V3/C/consC	Binding_V1V2/C/ZM109/1FD6	-0.75	<0.0001	<0.0001
	Binding_ V3/C/consC	ADCP_cV2/AE/92TH023	-0.51	0.0003	0.0008
	Binding_ V3/C/consC	Binding_cV2/C/ZM109	-0.46	0.0015	0.0028
	Binding_ gp120/C/ZM53	C1q_V1V2/C/ZM109/1FD6	-0.46	0.0017	0.0032
	Binding_ gp120/C/ZM53	ADCP_V1V2/B/YU2/1FD6	-0.47	0.0012	0.0024
	Binding_ gp120/C/ZM53	ADCP_cV2/C/1086	-0.46	0.0019	0.0036
	Binding_ gp120/C/ZM53	Binding_V1V2/AE/A244/1FD6	-0.37	0.0120	0.0181
	Binding_ gp120/C/ZM53	Binding_V2/AA190/209/AE/A244	-0.42	0.0038	0.0062
	Binding_ gp120/C/ZM53	Binding_V1V2/B/YU2/1FD6	-0.49	0.0006	0.0012
	Binding_ gp120/C/ZM53	C1q_V1V2/B/YU2/1FD6	-0.54	0.0001	0.0004
	Binding_ gp120/C/ZM53	Binding_V1V2/A/MG505/1FD6	-0.51	0.0003	0.0008
	Binding_ gp120/C/ZM53	ADCP_V1V2/C/ZM109/1FD6	-0.53	0.0002	0.0005
	Binding_ gp120/C/ZM53	Binding_V1V2/C/ZM109/1FD6	-0.48	0.0008	0.0017
	Binding_ gp120/C/ZM53	ADCP_cV2/AE/92TH023	-0.40	0.0071	0.0112
	Binding_ gp120/C/ZM53	Binding_cV2/C/ZM109	-0.20	0.1960	0.2253
	Binding_ gp120/A/MG505	C1q_V1V2/C/ZM109/1FD6	-0.64	<0.0001	<0.0001
	Binding_ gp120/A/MG505	ADCP_V1V2/B/YU2/1FD6	-0.65	<0.0001	<0.0001
	Binding_ gp120/A/MG505	ADCP_cV2/C/1086	-0.70	<0.0001	<0.0001
	Binding_ gp120/A/MG505	Binding_V1V2/AE/A244/1FD6	-0.60	<0.0001	<0.0001
	Binding_ gp120/A/MG505	Binding_V2/AA190/209/AE/A244	-0.65	<0.0001	<0.0001
	Binding_ gp120/A/MG505	Binding_V1V2/B/YU2/1FD6	-0.72	<0.0001	<0.0001
	Binding_ gp120/A/MG505	C1q_V1V2/B/YU2/1FD6	-0.75	<0.0001	<0.0001
	Binding_ gp120/A/MG505	Binding_V1V2/A/MG505/1FD6	-0.73	<0.0001	<0.0001
	Binding_ gp120/A/MG505	ADCP_V1V2/C/ZM109/1FD6	-0.73	<0.0001	<0.0001
	Binding_ gp120/A/MG505	Binding_V1V2/C/ZM109/1FD6	-0.70	<0.0001	<0.0001
	Binding_ gp120/A/MG505	ADCP_cV2/AE/92TH023	-0.54	0.0001	0.0003
	Binding_ gp120/A/MG505	Binding_cV2/C/ZM109	-0.38	0.0110	0.0169

Supplementary Figure S8. Statistics of correlations between different humoral immune responses in HIV-1 Env immunized or SHIV-infected NHPs. Correlation statistics are shown for clusters 1 and 4 of Figure 6. For each correlation, *r*, *p* values, and adjusted *p* values are displayed. All significant results (p <0.05) are highlighted in red.

Supplementary Figure S9

THP-1 cells

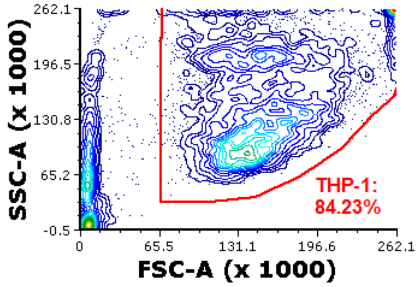

Singlets

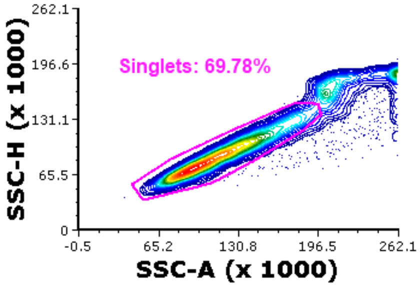

Bead+ THP-1 cells

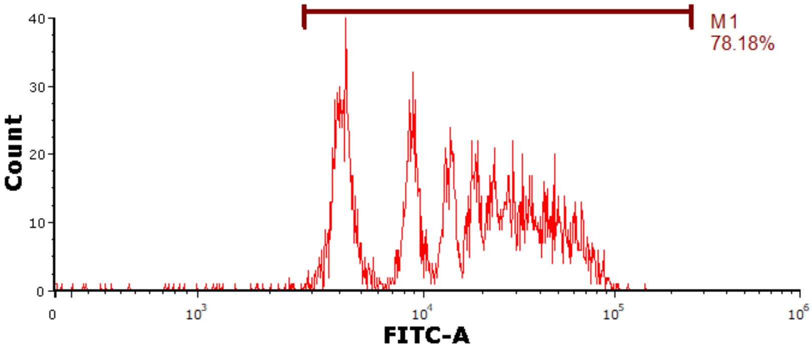

ADCP score = % FITC positive cells x MFI

Gate	# of events	% of gated cells
None	7970	100.00
THP-1 cells	6713	84.23
Singlets	4684	58.77
Bead+ THP-1-FITC	3662	45.95

Supplementary Figure S9. Gating strategy used for flow cytometry analysis. Representative FACS plots and histogram show gating strategy for measuring beads phagocytosed by THP-1 cells and calculation of the ADCP score.

Supplementary Table SI. Reactivity of V2p mAb CAP228-16H with peptides and V1V2-1FD6 scaffolds

V1V2 from HIV strain	Clade	V2p epitope sequence	Reactivity of CAP228-16H with peptide	Reactivity of CAP228-16H with V1V2-1FD6 scaffolds	Reference
1086	C	ELKDKKHKVHALFYKLDVV	+++	n.d.	Figure 1a
92TH023/A244	AE	ELRDKKQKVHALFYKLDIV	+++	+++	Figure 1a
CAP45.2.00.G3*	C	ELRDKKQKAYALFYRPDVV	+++	n.d.	16
CAP225.3.11.c*	C	EIRDKKQEVRALFYRSDIV	n.d.	+++	16
CAP200.3.8.1	C	EIRDKKQKAYALFYRPDIV	n.d.	+++	16
CAP229.2.00.CON	C	EIRDKKKQVYALFYKPDIV	n.d.	+++	16
CAP256.2.00.C7	C	EVRDKQKKEYALFYRLDLV	n.d.	-	16
CAP256.206sp.032.c9	C	ELRDKKKKEYALFYRLDIV	n.d.	-	16
CAP65.2.00.con	C	ELRDKKQKAYALFYRPDVV	n.d.	-	16
ZM109	C	DVKDRKQKVNATFYDLDIV	+++	-	Figure 1a
YU2	B	SIRDKVQKEYALFYNLDVV	-	-	Figure 1a
MG505	A	ELRDKKQKVYSLFYRLDVI	+++	-	Figure 1a
SHIV-1157ipEL-p	C	GIRDKKQKVNALFYRLDIT	n.d.	-	96
SHIV-1157ipd3N4	C	EIRDKKQKVYALFYRLDIT	n.d.	-	96

*Reagents with which CAP228-16H was crystallized; n.d., not done. Data from Wibmer et al. (16) were generated by ELISA; data from Figure 1a were generated with a multiplex bead binding assay.
Red residues: conserved amino acid substitutions; Blue: non-conserved amino acid substitutions; Green: major charge changes
Underlined: V2 residue K169: critical residue targeted by V2 Abs in RV144
