# Supplementary material for: Differential V2-directed antibody responses in non-human primates infected with SHIVs or immunized with diverse HIV vaccines
Source: Nat Commun. 2022 Feb 16;13:903. doi: 10.1038/s41467-022-28450-1 (PMC8850611; doi:10.1038/s41467-022-28450-1)
Supplement: Supplementary file 3 — Reporting Summary [file 41467_2022_28450_MOESM3_ESM.pdf]

nature portfolio

Corresponding author(s): Susan Zolla-Pazner, Ph.D.

Last updated by author(s): Dec 17, 2021

Reporting Summary

Nature Portfolio wishes to improve the reproducibility of the work that we publish. This form provides structure for consistency and transparency in reporting. For further information on Nature Portfolio policies, see our Editorial Policies and the Editorial Policy Checklist.

Statistics

For all statistical analyses, confirm that the following items are present in the figure legend, table legend, main text, or Methods section.

n/a	Confirmed	
☐	☒	The exact sample size (*n*) for each experimental group/condition, given as a discrete number and unit of measurement
☐	☒	A statement on whether measurements were taken from distinct samples or whether the same sample was measured repeatedly
☐	☒	The statistical test(s) used AND whether they are one- or two-sided<br>*Only common tests should be described solely by name; describe more complex techniques in the Methods section.*
☒	☐	A description of all covariates tested
☐	☒	A description of any assumptions or corrections, such as tests of normality and adjustment for multiple comparisons
☐	☒	A full description of the statistical parameters including central tendency (e.g. means) or other basic estimates (e.g. regression coefficient) AND variation (e.g. standard deviation) or associated estimates of uncertainty (e.g. confidence intervals)
☐	☒	For null hypothesis testing, the test statistic (e.g. *F*, *t*, *r*) with confidence intervals, effect sizes, degrees of freedom and *P* value noted<br>*Give P values as exact values whenever suitable.*
☒	☐	For Bayesian analysis, information on the choice of priors and Markov chain Monte Carlo settings
☐	☒	For hierarchical and complex designs, identification of the appropriate level for tests and full reporting of outcomes
☐	☒	Estimates of effect sizes (e.g. Cohen's *d*, Pearson's *r*), indicating how they were calculated

Our web collection on statistics for biologists contains articles on many of the points above.

Software and code

Policy information about availability of computer code

Data collection	xPONENT 4.2 software for Luminex (Austin, TX); CD-FIT program (http://www.ruppweb.org/Xray/comp/cdfit.htm); Gen5 software version 3.08 for BioTek microplate reader (Winooski, VT); BD FACSDiva Software version 8.0.2 for flow cytometry (Franklin Lakes, NJ)
Data analysis	GraphPad Prism 7.03 (San Diego, CA); Microsoft Excel 2013 (Redmond, WA); FCS Express 7 Flow Research Edition (De Novo Software, Pasadena, CA); R x64 version 4.0.2; (Indianapolis, IN) RStudio version 1.3.959 (Boston, MA)

For manuscripts utilizing custom algorithms or software that are central to the research but not yet described in published literature, software must be made available to editors and reviewers. We strongly encourage code deposition in a community repository (e.g. GitHub). See the Nature Portfolio guidelines for submitting code & software for further information.

Data

Policy information about availability of data

All manuscripts must include a data availability statement. This statement should provide the following information, where applicable:

- Accession codes, unique identifiers, or web links for publicly available datasets
- A description of any restrictions on data availability
- For clinical datasets or third party data, please ensure that the statement adheres to our policy

Luminex binding, C1q and ADCP data generated in this study are provided in the Supplementary Information/Source data.

Field-specific reporting

Please select the one below that is the best fit for your research. If you are not sure, read the appropriate sections before making your selection.

☒ Life sciences ☐ Behavioural & social sciences ☐ Ecological, evolutionary & environmental sciences

For a reference copy of the document with all sections, see nature.com/documents/nr-reporting-summary-flat.pdf

Life sciences study design

All studies must disclose on these points even when the disclosure is negative.

Sample size	Sample size was based on availability of NHP groups infected with different SHIV strains or immunized with different HIV Env vaccines.
Data exclusions	No data were excluded from the analyses.
Replication	All attempts at replication were successful. Luminex experiments: Plasma specimens screened at one dilution were run in duplicate. Additionally, specimens were titrated. All experiments were run at least twice. C1q assay: titrations were performed once. ADCP: titrations were performed at least twice.<br>All experiments: For inter-experimental standardization a monoclonal antibody pool or single mAb was used as a positive control in each experiment.
Randomization	Allocation of animals was random within each NHP study.
Blinding	Experiments were performed blinded.

Reporting for specific materials, systems and methods

We require information from authors about some types of materials, experimental systems and methods used in many studies. Here, indicate whether each material, system or method listed is relevant to your study. If you are not sure if a list item applies to your research, read the appropriate section before selecting a response.

Materials & experimental systems

n/a	Involved in the study
☐	☒ Antibodies
☐	☒ Eukaryotic cell lines
☒	☐ Palaeontology and archaeology
☐	☒ Animals and other organisms
☒	☐ Human research participants
☒	☐ Clinical data
☒	☐ Dual use research of concern

Methods

n/a	Involved in the study
☒	☐ ChIP-seq
☐	☒ Flow cytometry
☒	☐ MRI-based neuroimaging

Antibodies

Antibodies used	CH58, CH59, PGT145 and PG9 [NIH AIDS Reagents, Cat.No. 12550, Cat.No. 12551, Cat.No.12703, Cat.No.12149, (concentrations used varied dependent on the assay - see manuscript)]; CAP228-19F, CAP228-3D.1 and CAP228-16H provided by collaborator Lynn Morris (concentrations used varied dependent on the assay - see manuscript); Goat anti-monkey IgG (biotin) (Rockland, #617-106-012, used at 2 ug/ml); Rabbit anti-human IgG (biotin)(Abcam, #ab97158, used at 2 ug/ml); anti-C1q-HRP (Santa Cruz, clone 1A4, #sc-53544, used at 10 ug/ml); 830A, 2158, 1361, 1393A, 3869, 670 , 697-30D, 447-52D, 1331(A) and 3685 were generated from hybridoma cells and purified in the lab of Susan Zolla-Pazner (concentrations used varied dependent on the assay - see manuscript).
Validation	Monoclonal antibodies (mAbs) that had been generated in the lab of the corresponding author were validated through quality control assays (ELISAs against various HIV envelope antigens). Commercially obtained Abs were used per manufacturer's instructions and confirmed for reactivity for rhesus monkey samples or human samples.

Eukaryotic cell lines

Policy information about cell lines

Cell line source(s)	THP-1 cells (ATCC, TIB-202) and mAb-producing mouse x human hybridoma cell lines as described in references in the manuscript.
Authentication	Cell lines were commercially purchased (ATCC).

Mycoplasma contamination	All cell lines were tested regularly for mycoplasma contamination and were consistently negative.
Commonly misidentified lines (See ICLAC register)	NA

Animals and other organisms

Policy information about studies involving animals; ARRIVE guidelines recommended for reporting animal research

Laboratory animals	A total of 45 adult male (n=32) and female (n=13) rhesus macaques (M.mullata) ranging in age from 3 to 10 years of age were evaluated in this study. In all groups, animals were assigned and evenly distributed based on age, sex, and MHC compatibility needs for each group and/or project.
Wild animals	No wild animals were used.
Field-collected samples	No field-collected samples were used.
Ethics oversight	Macaque studies were performed at the Oregon National Primate Research Center (ONPRC) in Beaverton, OR, USA. The ONPRC is accredited by the American Association for the Accreditation of Laboratory Animal Care International, and adheres to the Guide for the Care and Use of Laboratory Animals and the U.S. Public Health Service Policy on the Humane Care and Use of Laboratory Animals. The Oregon Health & Science University (OHSU) West Campus Institutional Animal Care and Use Committee (IACUC) approved all macaque studies.

Note that full information on the approval of the study protocol must also be provided in the manuscript.

Flow Cytometry

Plots

Confirm that:

- [x] The axis labels state the marker and fluorochrome used (e.g. CD4-FITC).
- [x] The axis scales are clearly visible. Include numbers along axes only for bottom left plot of group (a 'group' is an analysis of identical markers).
- [x] All plots are contour plots with outliers or pseudocolor plots.
- [x] A numerical value for number of cells or percentage (with statistics) is provided.

Methodology

Sample preparation	Sample preparation is described in the methods.
Instrument	LSR II Fortessa (BD Bioscience; Franklin Lakes, NJ)
Software	collection of data: BD FACSDiva Software version 8.0.2 (Franklin Lakes, NJ); analysis of data: FCS Express 7 Flow Research Edition (De Novo Software, Pasadena, CA)
Cell population abundance	Indicative frequencies/abundance of populations are provided in Supplementary Information.
Gating strategy	In all experiments, populations were gated on FSC/SSC and single cells were retained using SSC-A/SSC-H. THP-1 cells that have phagocytosed beads were identified in the FITC channel.

- [x] Tick this box to confirm that a figure exemplifying the gating strategy is provided in the Supplementary Information.
